# An evolutionary approach to identify potentially protective B cell epitopes involved in naturally acquired immunity to malaria and the role of EBA-175 in protection amongst denizens of Bolifamba, Cameroon

**DOI:** 10.1186/s12936-016-1337-z

**Published:** 2016-05-20

**Authors:** Raymond B. Nyasa, Helen K. Kimbi, Denis Zofou, Jeremy D. DeBarry, Jessica C. Kissinger, Vincent P. K. Titanji

**Affiliations:** Biotechnology Unit, Faculty of Science, University of Buea, Buea, Cameroon; Department of Medical Laboratory Sciences, Faculty of Health Sciences, University of Bamenda, Bamenda, NWR Cameroon; Center for Tropical and Emerging Global Diseases, University of Georgia, Athens, USA; Department of Genetics, University of Georgia, Athens, USA; Institute of Bioinformatics, University of Georgia, Athens, USA; Cameroon Christian University, PO Box 5, Bali, NW Region Cameroon

**Keywords:** Natural acquired immunity, Lineage-specific, Hypothetical genes, EBA-175, Duration of stay, Malaria, Cameroon

## Abstract

**Background:**

The search for a vaccine against malaria caused by *Plasmodium falciparum* has lasted for more than 100 years, with considerable progress in the identification of a number of vaccine candidates. The post-genomic era offers new opportunities for an expedited search using rational vaccine design and prioritization of key B-cell epitopes involved in natural acquired immunity.

**Methods:**

Malaria vaccine candidate genes that have reached clinical trial were searched on an evolutionary relationship tree, to determine their level of lineage-specificity. Ten other genes with similar protein features and level of lineage specificity to the vaccine candidates were randomly selected, and computationally evaluated for the presence of B-cell epitopes. The protein fragment with maximum probability of putative epitopes were synthesized and used in an ELISA experiment to determine the presence of antibodies to these peptides, in the serum of malaria patients and healthy malaria uninfected inhabitants from a malaria endemic region (Bolifamba), alongside with a vaccine candidate EBA-175.

**Results:**

Two peptide fragments of 25 and 30 amino acid length from PF3D7_1233400 and PF3D7_1437500 respectively, coded as PF4-123 and PF4-143 were shown to contain B-cell epitope(s). Total IgG antibodies to these peptides were not significantly different between sick and healthy participants, but cytophilic antibodies to these peptides were significantly higher in healthy participants (p < 0.03). Total IgG to the vaccine candidate EBA-175 was significantly higher in sick participants than in healthy participants, likewise cytophilic antibodies (p < 0.04). Antibodies to the peptides PF4-123 and PF4-143 correlated negatively (p = 0.025 and 0.008 and r = −0.291 and −0.345, respectively) to parasite load. Total IgG antibodies to EBA-175 showed a negative correlation to parasite load (r = −0.144), which was not significant (p = 0.276). Duration of stay in Bolifamba also negatively correlated with parasite load (p = 0.026, r = −0.419) and total IgG to PF4-143 was significantly associated with prolonged duration of stay in the locality of Bolifamba, Cameroon (p = 0.006, r = 0.361).

**Conclusions:**

The present study has identified two genes PF3D7_1233400 and PF3D7_1437500 containing peptide fragment (PF4-123 and PF4-143) with B-cell epitopes that are correlated with naturally acquired immunity to malaria. A pipeline has been developed for rapid identification of other B-cell epitopes involved in naturally acquired immunity.

## Background

The global burden of malaria is still alarming despite wide spread use of anti-malarial drugs, treated bed nets and indoor residual spraying. In 2014, 3.3 billion people were still at risk of malaria infection and 584,000 people died of malaria in 2013 [1]. Unfortunately, there is no licensed vaccine against malaria approved by World Health Organization. Evidence for the feasibility of a malaria vaccine involving an antibody response stems from the fact that passive transfer of hyperimmune serum or purified immunoglobulins from people with life-long exposure to endemic malaria has been shown to decrease blood stage parasitaemia and resolve symptoms in malaria patients [2, 3]. Decades of research in the pre-genomic era using diverse approaches led to the identification of a handful of antigens implicated in immunity to malaria as vaccine candidates [4]. The arrival of the post-genomic era has given rise to immunomics, which offers a rational and systematic approach to vaccine target selection [4]. However, there remains no efficient method or algorithm to effectively analyse genomic, proteomic, and transcriptomic data, and to select which antigens are essential for protective immune response [4]. It has been shown that lineage-specific genes, formerly called orphan genes, are mostly putative surface antigens, rich in signal peptides and transmembrane domains, and are probably important in *Plasmodium* adaptation to its host [5]. Consequently these proteins are a source of potential vaccine targets, however this hypothesis needs to be validated for exploitation in rational vaccine design.

Current malaria vaccine candidates in clinical trials face the serious problem of being polymorphic [6]. To address this problem, the next generation of vaccines will probably be composed of chimeric molecules, or a combination of conserved proteins capable of eliciting protective immune responses against *Plasmodiumfalciparum*. Naturally acquired immunity to malaria is known to increase with age and duration of stay in an endemic region [7]. Children below 5 years of age, have mostly no naturally acquired immunity to malaria [7] and, therefore, account for the highest number of victims of malaria morbidity and mortality [1]. Thus, it is logical that serum antibodies from adults in an endemic region will recognize protein fragments involved in naturally acquired immunity to malaria relative to those observed in children. Cytophilic antibodies have been shown to positively correlate with protection from severe clinical manifestation of malaria [8] by opsonization [9]. Thus, a protein fragment involved in naturally acquired immunity to malaria will preferentially elicit cytophilic antibodies in healthy adults who demonstrate a lesser frequency of clinical malaria episodes, than in malaria patients. Previous work showed that total IgG and cytophilic antibodies to crude *P. falciparum* extract were significantly higher in adults than in children among inhabitants of Bolifamba, Cameroon [10]. In that study, it was proposed that peptides could be used to investigate this response. The current work specifically tests this hypothesis.

During *P. falciparum* infection, repeated cycles of parasite invasion of red blood cells (RBCs) quickly amplify blood-stage parasite load and aggravate malaria symptoms. EBA-175 is an antigen involved in merozoite invasion of RBCs which is known to bind to glycophorin A on human erythrocytes during the invasion process [11]. EBA-175 is a leading vaccine candidate and naturally produced antibodies to this antigen are predominantly cytophilic, and have been shown to correlate with protection from malaria [12, 13]. However, a study carried out in a cohort of Gambian children showed that naturally occuring total IgG and cytophilic IgG to EBA-175, measured prior to the malaria season, were not associated with protection during the malaria season [14]. In Cameroon, adults living in a malaria endemic village in Kumba (South West region) were shown to mount a very strong immune response to EBA, even higher than that of their counterparts in Brazil [15]. However, the age dependent nature of this immune response has not been demonstrated and correlation to parasite load has not been studied among Cameroonians. This study seeks to identify novel protective B-cell epitopes, and compare them to the leading vaccine candidate EBA-175.

## Methods

### Study area and participants

Blood samples used in this study were collected from Bolifamba, a malaria endemic locality on the eastern slope of Mount Cameroon, as reported in previous studies [16, 17]. The samples were collected between March and November 2014, from four groups of participants: 30 sick and 30 healthy children who were 5 years old or less, but not less than 1 year old, and 29 sick (who had one or more episodes of malaria within the past 1 year) and 28 healthy adults (who had not had malaria for at least a year) and were more than 14 years old. Sick participants were inhabitants of Bolifamba who were seeking medical attention for malaria at the Bolifamba Health Centre. Consenting participants from this group, were recruited into the study if they were within the age ranges, were diagnosed positive for malaria and had fever (temperature >37.5 °C). The healthy children and adult controls enrolled were randomly selected participants from the Bolifamba community, who were malaria negative by microscopy and had temperature ≤37.5, with an added criterion of admitting only adults who had not had malaria for at least a year. Information on the age, duration of stay in the community, and the last malaria episode was obtained from the adult participants.

### Ethical statement

The study was approved by the University of Buea Faculty of Health Sciences-Institutional Review Board, reference number 2013/144/UB/FHS/IRB. Administrative clearance was obtained from Ministry of Public Health Regional Delegation for the South West, reference number R11/MPH/SWR/RDPH/PS/108/263. All the participants who took part in the study were informed about the purpose of the study and signed the informed consent form. Consent of minors was obtained from their parents or guardians.

### Blood sample collection

Two ml of venous blood was collected from each participant and serum extracted from the blood was stored at −20 °C in 50 % glycerol, until used. Thick blood film smears were made on slides, air-dried and stained with 5 % Giemsa for quantification of parasite load by microscopy with the ×100 (oil immersion) objective.

### Bioinformatics

**Fig. 1:**
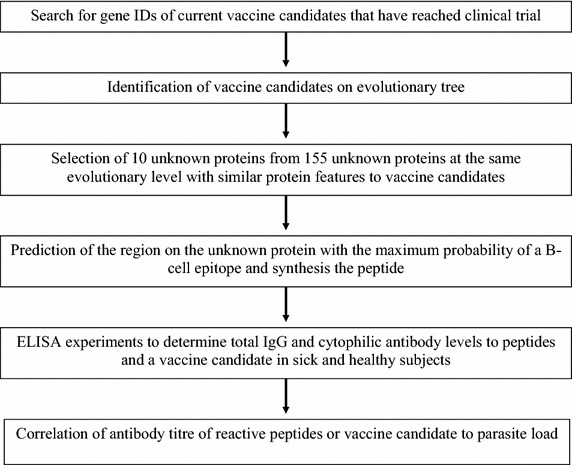
Flow chart of study design

**Fig. 2:**
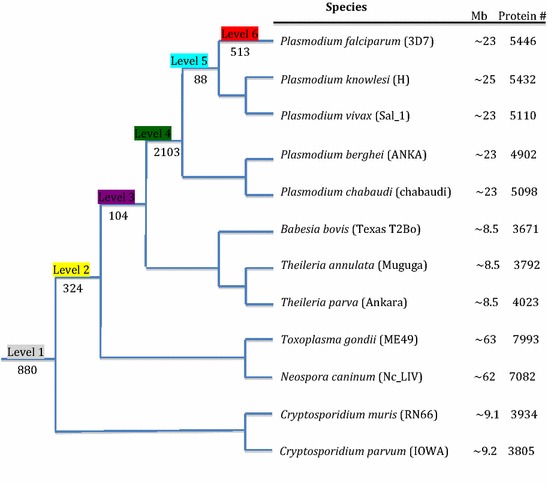
Species relationships and genome characteristics. A cladogram of investigated species with genome sizes and numbers of annotated protein-encoding genes. Strains follow species names. Numbered labels on the cladogram indicate the arbitrary levels and calculated number of ortholog clusters observed in descendants of the node. For example, level 1 contains ortholog clusters with members in all Apicomplexa (880 ortholog clusters). Level 5 contains ortholog clusters specific to *Plasmodium falciparum, Plasmodium knowlesi,* and *Plasmodium vivax* and not detected in the other apicomplexan species in the cladogram (88 ortholog groups). Protein # indicates the number of protein-encoding genes. Adapted from DeBarry and Kissinger, 2011 [18]

The study design shown in Fig. 1, was implemented as follows. The rainbow table reference list of global malaria vaccine projects was downloaded from the WHO website [18] and the research articles were visually scanned to obtain the PlasmoDB (version 9.3) gene IDs of the vaccine candidates, or the gene name was used in a search of PlasmoDB version 9.3 website [19] to obtain the gene IDs. This yielded a set of 22 genes. Each of these genes was then searched in the species relationship cladogram from DeBarry and Kissinger, 2011 [20] using the annotated protein-encoding genes of 12 apicomplexan species, to identify orthologous groups of genes (Fig. 2). In essence, orthologous gene clusters of all protein-encoding genes in twelve apicomplexan species were identified by a combination of WU-BLAST (version 2.2.6, E-value cutoff of 1 × 10^−30^) [21] for an all-by-all BLASTp similarity search and OrthoMCL (version 1.4) [22] with default parameters. The analysis was carried out on the University of Georgia rcluster and the output was parsed using customized PERL scripts. Orthologous clusters were searched to identify 155 single copy genes with evolutionary profiles similar to the 22 candidates. Of these, a sample of 10 was selected for further analysis. The genes encoding proteins with features similar to vaccine candidates (presence of transmembrane domain, and/or signal peptide), are annotated as part of the plasma-membrane, have no known B or T-cell epitopes, and have mass-spectrometry evidence of the existence of the protein as revealed by PlasmoDB version 9.3 [19].

The set of 10 genes was then assessed for B-cell epitopes. The Bepipred Linear Epitope Prediction method in Immune Epitope Data Base (IEDB) makes use of hidden Markov model and a propensity scale was used to predict B-cell epitopes on the selected protein sequences [23]. Only the region with the maximum probability of containing a B-cell epitope was selected. The presence of B-cell epitopes in the selected region was confirmed using Parker Hydrophilicity Prediction [24], Karplus and Schulz flexibility scale [25], Emini surface accessibility scale [26], and Chou and Fasman beta turn prediction [27], with progressive expansion of the predicted region to a maximum of 30 amino acids based on the output of the confirmatory methods in the prediction of the extent of B-cell epitopes on the selected region. The selected peptide sequences were synthesized by GL Biochem, Shanghai, China, at a purity of greater than 95 %. Upon arrival, the peptides were reconstituted to a concentration of 1 mg/mL in PBS containing 0.03 % of sodium azide and stored in aliquots of 50 μL at −20 °C until used.

### Measurement of antigen-specific antibody responses

Checkerboard titration was used to determine the working antigen concentration and serum dilution for each antigen prior to screening of the entire serum sample collection. The vaccine candidate EBA-175 was obtained through MR4/BEI Resources, NIAID, NIH: EBA-175 RII-NG, MRA-1162, and used at a concentration of 4 μg/mL in carbonate buffer, pH 9.6 to coat 96-well, flat bottom, high-binding polystyrene ELISA plates (Costar, Corning Incorporated, Corning, NY) with 100 μL/well, overnight (18 h) at 4 °C. Blocking was done with 0.2 % casein in PBS-Tween20 (0.05 %) at 200 μL/well for 1.5 h at room temperature. Washing was performed three times between each step using PBS-T, followed by incubation with serum in duplicate at 1:100 dilution in 1 % non-fat skimmed milk-PBS-T at 37 °C. Total IgG antibodies to EBA175 was detected by incubation with 1:10,000 dilution of goat-anti-human IgG (Fc specific)-peroxidase (Sigma-Aldrich, St. Louis, MO) in 1 % non-fat skimmed milk-PBS-T for 1.5 h at room temperature. Plates were developed in the dark with 1 mg/mL o-phenylene diamine (Sigma-Aldrich, St. Louis, MO) in 70 mM citrate-phosphate buffer, pH 4.2 containing H_2_O_2_ for 30 min in the dark and the optical density was read at 450 nm with an Emax Precision Microplate ELISA reader (Molecular Devices, California, USA). Five European naïve sera were included in the experiments as negative controls.

Cytophilic antibodies (IgG1 and IgG3) are important mediators of malaria parasite clearance in humans [28]. IgG1 and IgG3 were measured using the same procedure as above, at a serum dilution of 1:40. Subclasses IgG1 and IgG3 to EBA 175 were detected by incubation with monoclonal mouse anti-human IgG1 and monoclonal mouse anti-human IgG3 antibodies (Sigma–Aldrich, St. Louis, MO), respectively at 1:10,000 dilution for 2.5 h at room temperature, followed by incubation with 1:3000 dilution of anti-mouse IgG (Fab)-Peroxidase (Sigma-Aldrich, St. Louis, MO) at room temperature for 1.5 h. Both monoclonal mouse anti-human IgG1, IgG3 and anti-mouse IgG (Fab)-Peroxidase were diluted in 1 % skimmed milk-PBS-T.

The peptide ELISA was carried out using the same procedure as above, with some modifications. Coating was done with 4 μg/mL of peptide in carbonate buffer, at 37 °C overnight, while the ELISA plates were being rotated at 2.8 rpm using an adapted device placed inside the incubator (Heraeus, Hanau, Germany). Blocking was performed for 1 h using 150 μL of 0.2 % casein in PBS-T. Serum was diluted at 1:50 in 1 % nonfat skimmed milk-PBS-T and incubated for 2 h 20 min at 37 °C. The procedure for measurement of IgG1 and IgG3 antibody subclasses to the peptides were as in the measurement of EBA-175 IgG1 and IgG3 antibody subclasses with the same modifications as in measurement of total antibodies to the peptides, and the serum was used at 1:30 dilution. All peptides that did not show an antigen concentration dependent and serum dilution dependent optical density during the checkerboard titration were eliminated from the ELISA experiments.

### Statistical analysis

The data were analysed using IBM SPSS statistics version 21.0. The Mann–Whitney U non-parametric test was used to compare differences in antibody responses between study groups and the Spearman’s rank correlation test was used to test for significance of correlation between log of parasite load and antibody responses or duration of stay in Bolifamba. The data was graphically presented using SPSS and Microsoft Excel.

## Results

### Bioinformatics

Evolutionary analysis of the vaccine candidates that have reached clinical trial showed that protective immunity to malaria was strongly genus- and species-specific (Table 1). The majority of the vaccine candidates are proteins shared by most members of the genus *Plasmodium* (45.5 %) or proteins that are unique to *P. falciparum* (45.5 %). Ten additional *P. falciparum* genes with unknown functions, similar evolutionary profiles and evidence of a putative surface localization, (i.e., the gene product description containing the word membrane and/or annotation as part of plasma-membrane, a signal peptide and/or transmembrane domain) were identified. Of these, two genes were selected from the set of genes unique to *P. falciparum*, four from the set of genes unique to *Plasmodium* genus and four from the set of genes common to *Plasmodium*, Piroplasms, *Toxoplasma* and *Neospora* (Levels 6, 4 and 2, respectively, Table 1; Fig. 2) for a total of 10 genes, Table 2. A search of each of these 10 protein sequences for the region with the highest probability of containing a B-cell epitope was performed using the Bipred algorithm which yielded results in a range of probability from 0.5 to 0.72. Among the 10 peptides synthesized from these proteins, two showed a concentration of peptide or serum dilution dependent response in the checkerboard titration (PF4-123 and PF4-143) and they were among the four proteins with the highest probability of containing B-cell epitopes of 0.7–0.72 (Table 2). Subsequent analyses were limited to PF4-123 and PF4-143 and the known vaccine candidate EBA-175 for comparison.

**Table 1:** Evolutionary relationship levels of malaria vaccine candidates that have reached clinical trials

Level 1	Level 2	Level 3	Level 4	Level 5	Level 6
0	PF3D7_0603400 (TEX1)	0	PF3D7_1216600 (CelTOS)	0	PF3D7_1200600 (VAR2CSA)
	PF3D7_1133400 (AMA1)		PF3D7_1031000 (Pfs25)		PF3D7_1035300 (GLURP)
			PF3D7_1346700 (P48/45)		PF3D7_1035400 (MSP3)
			PF3D7_1335900 (TRAP)		PF3D7_1036400 (LSA1)
			PF3D7_0930300 (MSP1)		PF3D7_0206800 (MSP2)
			PF3D7_0207600 (SERA5)		PF3D7_1121600 (EXP1)
			PF3D7_0404500 (P52)		PF3D7_0406200 (Pfs16)
			PF3D7_0404400 (P36)		PF3D7_0102200 (RESA)
			PF3D7_0304600 (CSP)		PF3D7_0702300 (STARP)
			PF3D7_0731500 (EBA175)		PF3D7_0220000 (LSA3)

Levels correspond to phylogenetic relationships defined in Fig. 2. Genes are identified by their official ID number and protein name in parentheses

**Table 2:** Summary of information and bioinformatics analyses of the 10 selected peptides

Level	Gene ID	Product description	Annotated GO component term	Location aa position	Code	Peptide aa sequence	Length	Maximum probability of B-cell epitope by Bepipred
6	PF3D7_1112000	Conserved Plasmodium protein, unknown function	Integral to membrane, plasma membrane	54–72	PF6-111	KFNYDPFYSNWEKKNIQDS	19	0.52
6	PF3D7_0601900	Conserved Plasmodium protein, unknown function	Maurer’s cleft	76–98	PF6-060	MSKHYEDDDDDDDYQPPRHSSLP	23	0.68
4	PF3D7_1313500	Conserved Plasmodium membrane protein, unknown function	extracellular region, membrane	740–769	PF4-131	KSHHKNIHNNNTVEYNSEEDGNSKSKLSKD	30	0.7
4	PF3D7_1233400	Conserved Plasmodium membrane protein, unknown function	Cell surface, extracellular region	489–513	PF4-123	RKKIYTHKTTRKKHKDNPDYEKALL	25	0.71
4	PF3D7_1437500	Conserved Plasmodium membrane protein, unknown function	Integral to membrane, plasma membrane	7–36	PF4-143	VKIDNGESDEYNSTNQSPRKLNDSSGLSKK	30	0.75
4	PF3D7_1138200	Conserved Plasmodium protein, unknown function	Integral to membrane, plasma membrane	7–23	PF4-113	ICGRPLRNGGTAPLIYN	17	0.58
2	PF3D7_0209600	Transporter, putative	Integral to membrane	6–27	PF2-020	RSSVTRTSNEESNEDDKNCVNV	22	0.561
2	PF3D7_1471200	Inorganic anion exchanger, inorganic anion antiporter (SulP)	Integral to plasma membrane, membrane	65–84	PF2-147	IKWGWGFTNTPKETSKYYIN	20	0.72
2	PF3D7_1250200	Conserved Plasmodium membrane protein, unknown function	Apicoplast, integral to membrane, membrane	445–474	PF2-125	DKDDNKEDDNNDDDNNDNHHNNDDNNDDHH	30	0.65
2	PF3D7_1125000	Conserved Plasmodium protein, unknown function	Apicoplast, plasma membrane	129–148	PF2-112	FNVEEMGTGKTDDIHTPIEV	20	0.6

Level is with respect to Fig. 2. *aa* amino acid, *Code* peptide fragment name

### Differential total IgG responses to EBA-175, PF4-123 and PF4-143

**Fig. 3:**
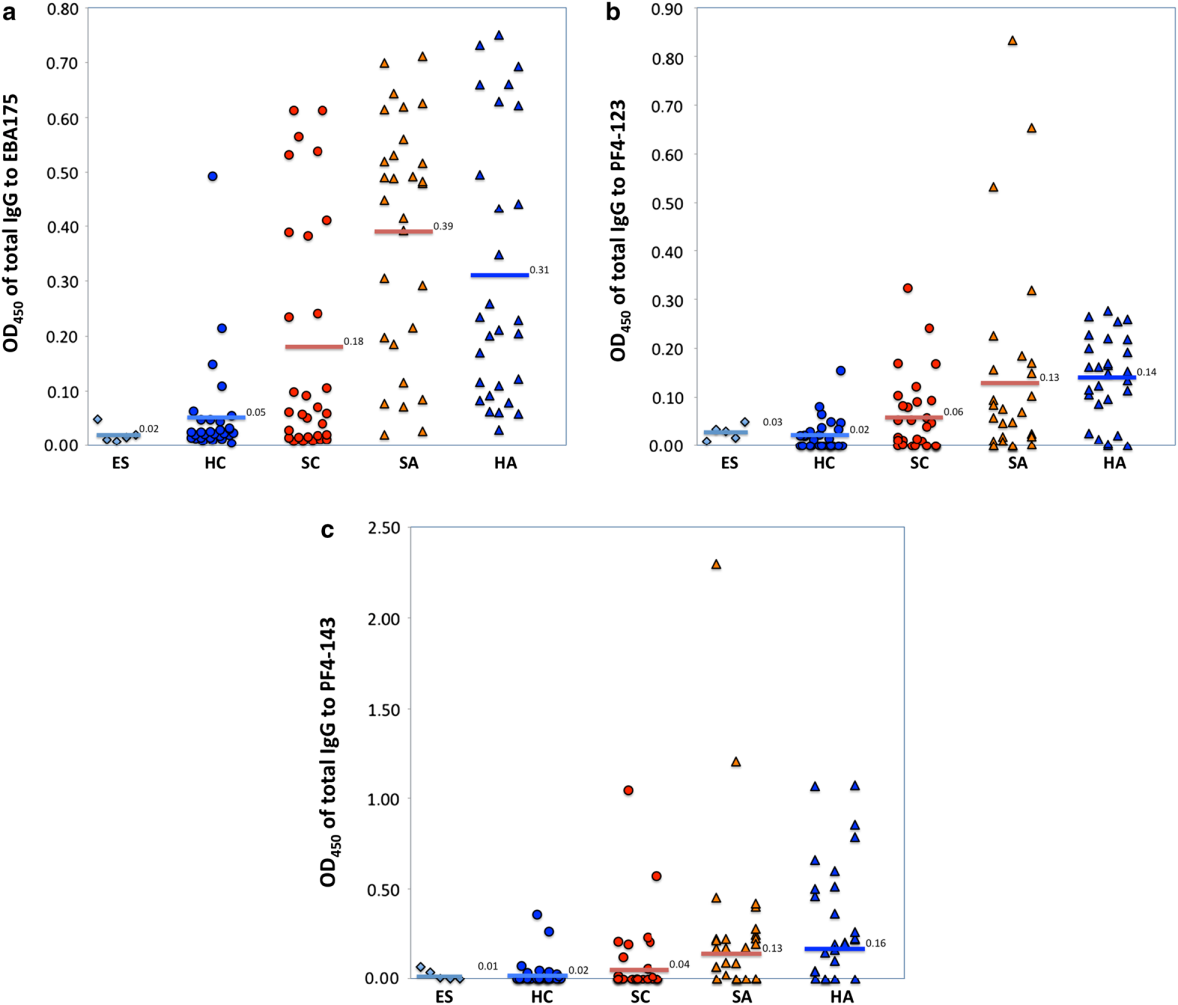
Mean optical densities (ODs) for total IgG; **a** EBA-175, **b** PF4-123, **c** PF4-143. The *bars* represent the arithmetic mean OD values of European sera (ES), healthy children (HC), sick children (SC), sick adults (SA), and healthy adults (HA). More details are under materials and methods

Comparison of adults to children showed a strong age-dependent response, with adults having significantly higher (p < 0.01) antibody responses for each of the three antigens (EBA-175, PF4-123 and PF4-143) (Fig. 3). This age-dependent response was present for the subgroup of healthy participants, likewise sick participants (p < 0.01) with the exception of the antibody response amongst sick participants to PF4-123, which showed no significant difference between sick and healthy adults (p = 0.2).

Total IgG response to EBA-175 was significantly higher in sick participants compared to healthy participants (p = 0.04), and was higher among sick children compared to healthy children (p = 0.04), but there was no significant difference between sick adults and healthy adults (p = 0.3). Total IgG to PF4-123 showed no significant difference between healthy and sick participants (p = 0.99). However, healthy adults had a significantly higher antibody response than sick adults (p = 0.04), contrary to what was obtained in the children’s subgroup, where sick children had a significantly higher antibody response compared to healthy children (p = 0.03). Total IgG response to PF4-143 was not significantly associated with health status (p > 0.1) whether in adults or children.

### Differential IgG1 antibody subclass response to EBA-175, PF4-123 and PF4-143

**Fig. 4:**
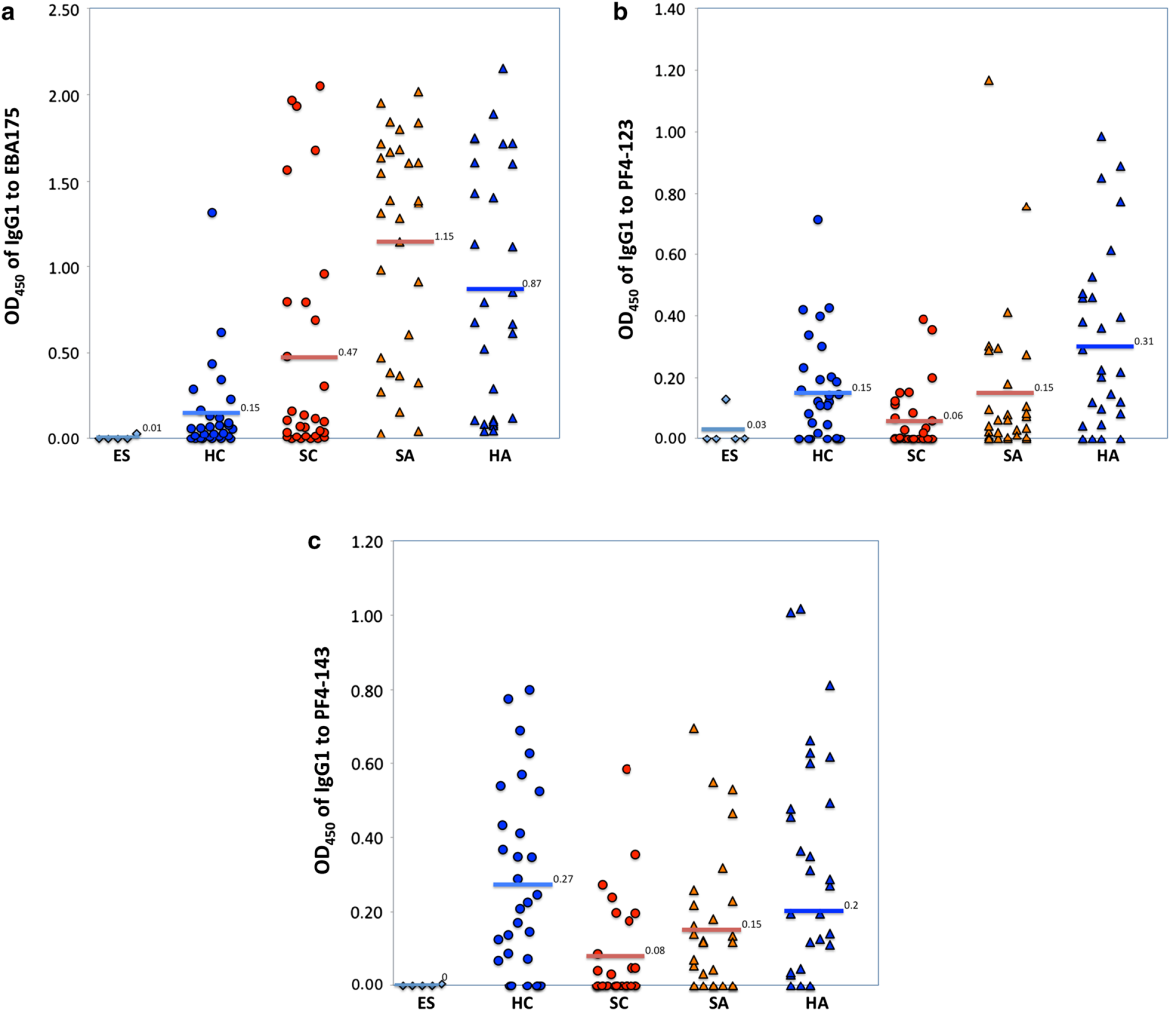
Mean optical densities (ODs) for IgG1 antibody subclass to; **a** EBA-175, **b** PF4-123, **c** PF4-143. The *bars* represent the arithmetic mean OD values of European sera (ES), healthy children (HC), sick children (SC), sick adults (SA), and healthy adults (HA)

IgG1 subclass response to EBA-175 and PF4-123 was significantly higher in adults than in children (p < 0.01), likewise, they were significantly higher in healthy adults than in healthy children and in sick adults than in sick children (p < 0.05) (Fig. 4). However, IgG1 subclass response to PF4-143 was not significantly different between adults and children (p = 0.1) or between healthy adults and healthy children (p = 0.50), but was significantly higher among sick adults compared to sick children (p = 0.047).

IgG1 response to PF4-123 and PF4-143 were significantly higher in healthy participants than in sick participants (p < 0.01), likewise in healthy adults than in sick adults (p < 0.02) and in healthy children than in sick children (p < 0.02). Contrary to IgG1 response to EBA-175, which was significantly higher in sick participants than in healthy participants (p = 0.03), there was no significant difference between sick and healthy adults and between sick and healthy children.

### Differential IgG3 antibody subclass response to EBA-175, PF4-123 and PF4-143

**Fig. 5:**
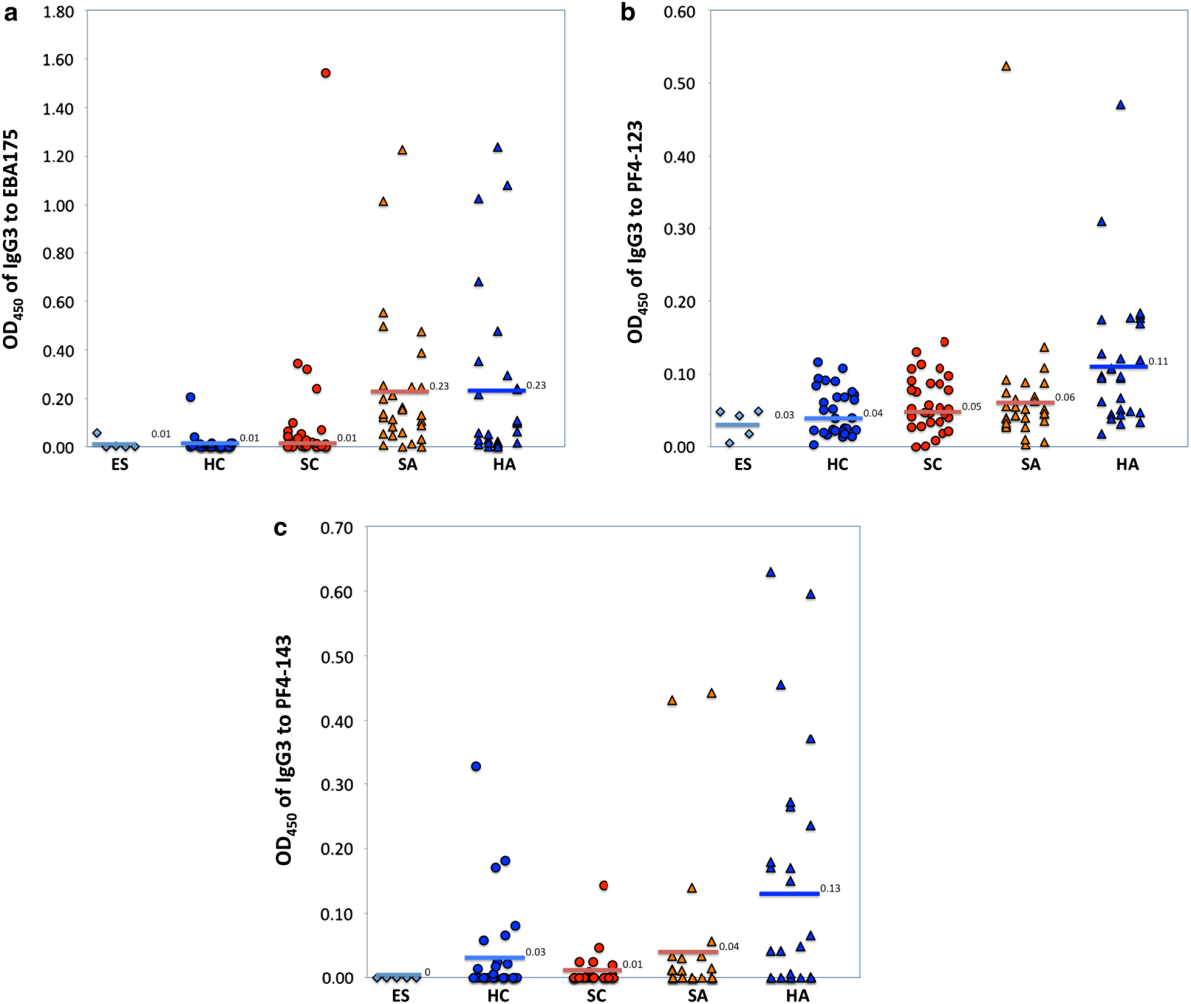
Mean optical densities (ODs) for IgG3 antibody subclass to; **a** EBA-175, **b** PF4-123, **c** PF4-143. The *bars* represent the arithmetic mean OD values of European sera (ES), healthy children (HC), sick children (SC), sick adults (SA), and healthy adults (HA)

IgG3 subclass response to all three antigens (EBA-175, PF4-123, and PF4-143) was significantly higher in adults than in children (p < 0.02), and in healthy adults than in healthy children (p < 0.021) (Fig. 5). However IgG3 subclass response to PF4-123 and PF4-143 was not significantly different between sick adults and sick children. IgG3 antibodies to EBA-175 were significantly higher among sick adults compared to sick children (p < 0.01).

IgG3 antibody subclass to EBA-175 was significantly higher in sick participants compared to healthy participants (p < 0.01) and in sick children compared to healthy children (p < 0.01), but there was no significant difference between sick adults and healthy adults. Contrary to EBA-175, PF4-143 was significantly higher in healthy participants, compared to sick participants (p = 0.013) and in healthy adults compared to sick adults (p = 0.023), however there was no significant difference between healthy and sick children. PF4-123 was not significantly different between healthy and sick participants, or between healthy children and sick children but it was significantly higher among healthy adults compared to sick adults (p < 0.01).

### Correlation of total antibody response to parasite load

**Fig. 6:**
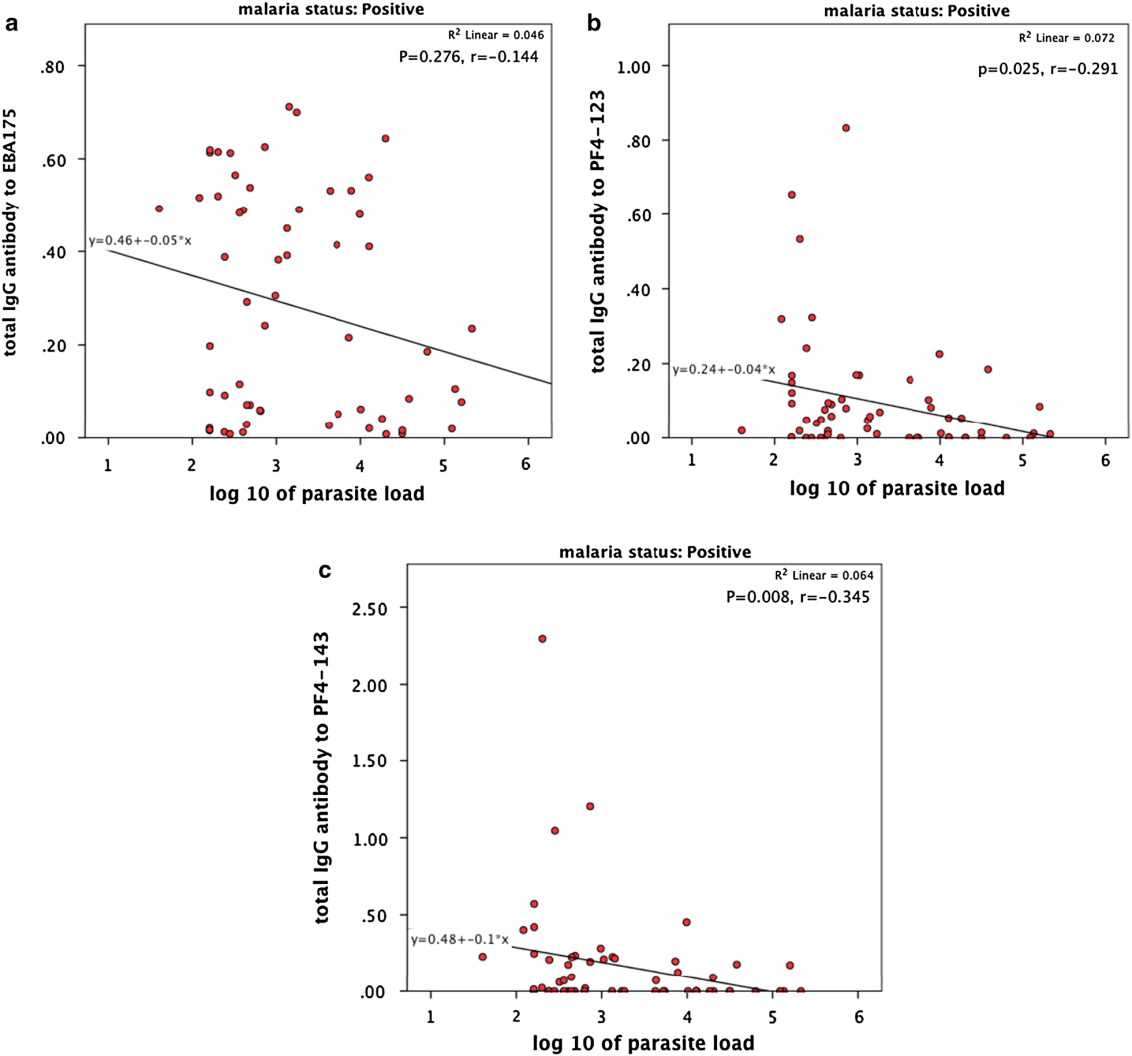
Correlation and regression equation of parasite load to total IgG antibodies to; **a** EBA-175, **b** PF4-123, **c** PF4-143. *r* Spearman’s rank correlation coefficient, *p* two-tailed level of significance

Total IgG antibody response to all antigens (EBA-175, PF4-123 and PF4-143) had a negative correlation to increasing parasite load (Fig. 6). Notably PF4-143 had the strongest negative correlation to increasing parasite load (p < 0.01) followed by PF4-123 (p < 0.05) and EBA-175 had the least negative correlation to parasite load, which was not significant (p = 0.276).

### Duration of residency in Bolifamba

**Fig. 7:**
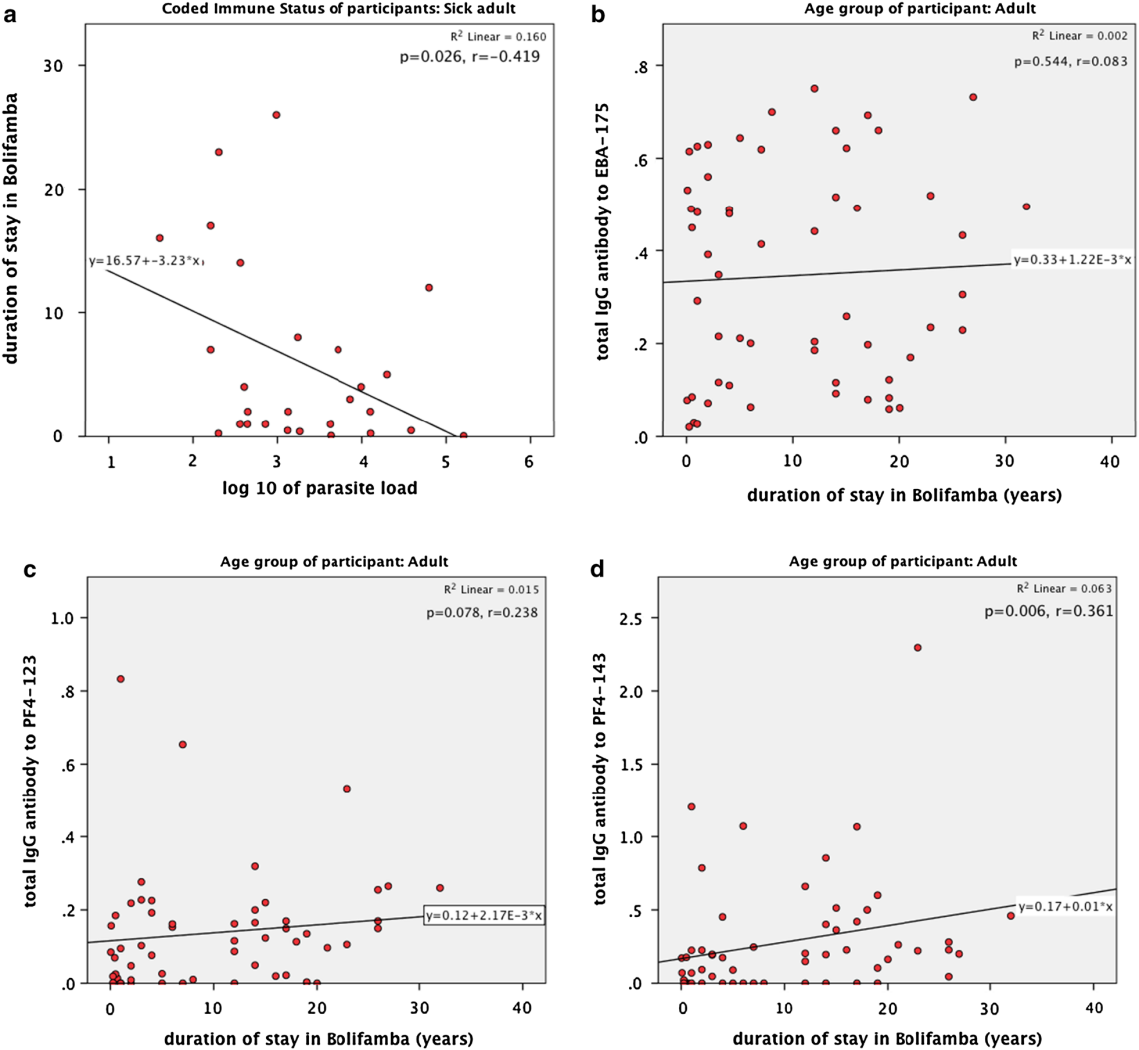
Correlation and regression equation of duration of stay in Bolifamba to parasite load (**a**) and total IgG antibodies to; (**b**) EBA-175, (**c**) PF4-123, (**d**) PF4-143. *r* Spearman’s rank correlation coefficient, *p* two-tailed level of significance

Generally, healthy adult participants in this study had lived for a significantly longer period of time in Bolifamba than sick adult participants (p < 0.001). Among the sick adults, duration of stay in Bolifamba negatively correlated with parasite load (p < 0.01, r = −0.419) (Fig. 7). Increasing duration of stay in Bolifamba had a positive correlation with antibody response for all three antigens (EBA-175, PF4-123 and PF4-143), however, only PF4-143 had a significant correlation to duration of stay in Bolifamba (p < 0.01, r = 0.361).

## Discussion

Bioinformatics analysis of current vaccine candidates revealed that they are strongly species-specific. This is in agreement with results from the old practice of using induced malaria as a therapy for neurosyphilis, in which second and third inoculations with homologous or heterologous strains of the same species of *Plasmodium* led to reduced symptoms and decreased parasite density [29]. The genus-specific nature of protective immune response to vaccine candidates corroborates with the observation that orthologous proteins that were protective in *P. falciparum* were also protective in *P. vivax* [30]. This is an indication that vaccine research in malaria has been making progress in the right direction. However, limited success is being achieved because close to 50 % of the antigens, which have reached clinical trial are coded by species-specific genes. This group includes paralogous gene families, which introduce issues related to polymorphisms, as is the case with vaccine candidates currently under clinical trial [6]. The other ~50 % of vaccine candidates that are genus-specific, are comprised of antigens from paralogous gene families such as the erythrocyte binding family (e.g. EBA-175), merozoite surface protein family (e.g. MSP1) and others. These also contain polymorphism issues. This study was limited to unknown, single-copy antigens, with similar properties and evolutionary relationships as current vaccine candidates and the data suggest that the two identified proteins (PF3D7_1233400, [PF4-123] and PF3D7_1437500, [PF4-143]) could be better vaccine candidates than EBA-175.

In this study, there was no significant difference in the total IgG response to EBA-175, between sick adults and healthy adults. This is in agreement with previous work in Cameroon [15]. However, this work goes further to show that antibodies to EBA-175 are significantly higher in sick children when compared to healthy children. EBA-175, PF4-123, and PF4-143 all showed an age dependent response, in which adults had a significantly higher antibody response, compared to children, which is characteristic of naturally acquired immunity. The fact that PF4-123 and PF4-143 had no significant difference in total antibody IgG levels between sick and healthy participants and the significantly higher level of cytophilic antibodies (IgG1 and IgG3) in healthy participants, suggest that antibodies to these antigens are constitutively produced and may be necessary for the reduction of risk of contracting malaria in this geographical region. These immune responses are contrary to what obtained with EBA-175 where total IgG and cytophilic antibodies are significantly higher in sick participants when compared to healthy participants. The possible involvement of antibodies to PF4-123 and PF4-143 in protection against malaria is further buttressed by the significantly stronger negative correlation it had with parasite load. This is in agreement with the fact that antiparasite immunity confers protection against parasitaemia, which affects the density of parasite [7]. Total IgG to EBA-175 also showed a negative correlation to parasite load, but it was not significant. Thus, PF4-123 and PF4-143 may also be involved in anti-parasitic immunity to malaria.

The demonstration of possible malaria protective properties by antibodies to PF4-123 and PF4-143 in this study opens the gateway for assessing the feasibility of monoclonal antibody therapy to malaria. Likewise a cocktail of peptides which elicit protective antibodies to malaria might be used to immunize people and re-establish naturally acquired immunity to malaria, which is typically lost with progressive elimination of malaria in various localities [7]. This type of approach, if effective could avert the kind of catastrophic rebound of malaria that occurred in the highlands of Madagascar in the 1980s, in which more than 40,000 people lost their lives in malaria epidemics. Interventions in the Madagascar highlands to control the mosquito vector population had reduced exposure below a level capable of maintaining naturally acquired immunity and a collapse of epidemiological surveillance led to re-colonization by *Anopheles funestus,* which caused a series of malaria epidemics **[**31**]**. This work has also developed a pipeline to identify additional potentially protective B-cell epitopes against malaria. The approach can be applied to other infectious diseases.

## Conclusions

This work has identified two genes PF3D7_1233400 and PF3D7_1437500, which encode peptide fragments containing B-cell epitopes, PF4-123 and PF4-143, respectively, that are involved in naturally acquired immunity to malaria.

## Abbreviations

RBCs: red blood cells
WHO: World Health Organization

## References

[1] WHO. World Malaria Report. Geneva: World Health Organization; 2014. http://www.who.int/malaria/media/world_malaria_report_2014/en/. Accessed 24 Jul 2015.

[2] Cohen S, McGregor IA, Carrington S (1961). Gamma-globulin and acquired immunity to human malaria. Nature.

[3] Sabchareon A, Burnouf T, Ouattara D, Attanath P, Bouharoun-Tayoun H, Chantavanich P (1991). Parasitologic and clinical human response to immunoglobulin administration in *falciparum* malaria. Am J Trop Med Hyg.

[4] Doolan DL (2011). *Plasmodium* immunomics. Int J Parasitol.

[5] Kuo CH, Kissinger JC (2008). Consistent and contrasting properties of lineage-specific genes in the apicomplexan parasites *Plasmodium* and *Theileria*. BMC Evol Biol.

[6] Ouattara A, Takala-Harrison S, Thera MA, Coulibaly D, Niangaly A, Saye R (2013). Molecular basis of allele-specific efficacy of a blood-stage malaria vaccine: vaccine development implications. J Infect Dis.

[7] Doolan DL, Dobaño C, Baird JK (2009). Acquired immunity to malaria. Clin Microbiol Rev.

[8] Leoratti FMS, Durlacher RR, Lacerda MVG, Alecrim MG, Ferreira AW, Sanchez MCA (2008). Pattern of humoral immune response to *Plasmodium falciparum* blood stages in individuals presenting different clinical expressions of malaria. Malar J.

[9] Osier FH, Feng G, Boyle MJ, Langer C, Zhou J, Richards JS (2014). Opsonic phagocytosis of *Plasmodium falciparum* merozoites: mechanism in human immunity and a correlate of protection against malaria. BMC Med.

[10] Titanji VP, Tamu VD, Nkuo-Akenji TK, Joutchop AS (2002). Immunoglobulin G and subclass responses to *Plasmodium falciparum* antigens: a study in highly exposed Cameroonians. Clin Chem Lab Med.

[11] Sim BK, Chitnis CE, Wasniowska K, Hadley TJ, Miller LH (1994). Receptor and ligand domains for invasion of erythrocytes by *Plasmodium falciparum*. Science.

[12] Medeiros MM, Fotoran WL, Dalla Martha RC, Katsuragawa TH, Pereira da Silva LH, Wunderlich G (2013). Natural antibody response to *Plasmodium falciparum* merozoite antigens MSP5, MSP9 and EBA175 is associated to clinical protection in the Brazilian Amazon. BMC Infect Dis.

[13] Dobaño C, Quelhas D, Quintó L, Puyol L, Serra-Casas E, Mayor A (2012). Age-dependent IgG subclass responses to *Plasmodium falciparum* EBA-175 are differentially associated with incidence of malaria in Mozambican children. Clin Vaccine Immunol.

[14] Okenu MND, Riley ME, Bickle DQ, Agomo UP, Barbosa A, Daugherty RJ (2000). Analysis of human antibodies to erythrocyte binding antigen 175 of *Plasmodium falciparum*. Infect Immun.

[15] Ford L, Lobo CA, Rodriguez M, Zalis MG, Machado RL, Rossit AR (2007). Differential antibody responses to *Plasmodium falciparum* invasion ligand proteins in individuals living in malaria-endemic areas in Brazil and Cameroon. Am J Trop Med Hyg.

[16] Nkuo-Akenji T, Ntonifor NN, Ndukum MB, Abongwa EL, Nkwescheu A, Anong DN (2006). Environmental factors affecting malaria parasite prevalence in rural Bolifamba, South- West Cameroon. Afr J Health Sci.

[17] Akenji TN, Ntonifor NN, Kimbi HK, Abongwa EL, Ching JK, Ndukum MB (2005). The epidemiology of malaria in Bolifamba, a rural community on the eastern slopes of Mount Cameroon: seasonal variation in the parasitological indices of transmission. Ann Trop Med Parasitol.

[18] WHO. Immunization, vaccines and biologicals: Rainbow table reference list. Geneva: World Health Organization http://www.who.int/vaccine_research/links/Rainbow/en/index.html. Accessed 10 May 2013.

[19] PlasmoDB. http://plasmodb.org/plasmo/. Accessed 11 June 2013.

[20] DeBarry JD, Kissinger JC (2011). Jumbled genomes: missing Apicomplexan synteny. Mol Biol Evol.

[21] Altschul SF, Gish W, Miller W, Myers EW, Lipman DJ (1990). Basic local alignment search tool. J Mol Biol.

[22] Li L, Stoeckert CJ, Roos DS (2003). OrthoMCL: identification of ortholog groups for eukaryotic genomes. Genome Res.

[23] Larsen JE, Lund O, Nielsen M (2006). Improved method for predicting linear B-cell epitopes. Immunome Res.

[24] Parker JM, Guo D, Hodges RS (1986). New hydrophilicity scale derived from high-performance liquid chromatography peptide retention data: correlation of predicted surface residues with antigenicity and X-ray-derived accessible sites. Biochemistry.

[25] Karplus PA, Schulz GE (1985). Prediction of chain flexibility in proteins—a tool for the selection of peptide antigens. Naturwissenschafren.

[26] Emini EA, Hughes JV, Perlow DS, Boger J (1985). Induction of hepatitis A virus-neutralizing antibody by a virus-specific synthetic peptide. J Virol.

[27] Chou PY, Fasman GD (1978). Prediction of the secondary structure of proteins from their amino acid sequence. Adv Enzymol Relat Areas Mol Biol.

[28] Garraud O, Mahanty S, Perraut R (2003). Malaria-specific antibody subclasses in immune individuals: a key source of information for vaccine design. Trends Immunol.

[29] Jeffery GM (1966). Epidemiological significance of repeated infections with homologous and heterologous strains and species of *Plasmodium*. Bull World Health Organ.

[30] Ce spedes N, Habel C, Lopez-Perez M, Castellanos A, Kajava AV, Servis C (2014). *Plasmodium vivax* antigen discovery based on alpha- helical coiled coil protein motif. PLoS ONE.

[31] Romi R, Razaiarimanga MC, Raharimanga R, Rakotondraibe EM, Ranaivo LH, Pietra V (2002). Impact of the malaria control campaign (1993–1998) in the highlands of Madagascar: parasitological and entomological data. Am J Trop Med Hyg.

